# Experienced teammates increase productivity in remote work: Evidence from a full remote work company in Japan

**DOI:** 10.1371/journal.pone.0342730

**Published:** 2026-03-17

**Authors:** Hideaki Ishikura

**Affiliations:** Graduate School of Media and Governance, Keio University, Fujisawa, Kanagawa, Japan; International University - Vietnam National University Ho Chi Minh City, VIET NAM

## Abstract

This study examines peer effects among employees working fully remotely. We use panel data from a company that has operated with an entirely remote workforce since its inception and leverage as-if random assignment of new hires to teams as a quasi-natural experiment. We find no evidence that the average productivity of a worker’s teammates affects that worker’s own productivity. However, when team members are highly experienced, the productivity of employees on those teams increases by about 12.2%. In particular, employees with the shortest tenure see an increase in productivity of approximately 26.2%. Furthermore, this effect appears unrelated to the volume of communication within the team, suggesting that experienced teammates can have a positive influence even with minimal interaction, possibly through more efficient, targeted communication.

## Introduction

One of the major changes in work arrangements due to the COVID-19 pandemic is the rise of remote work; as of June 2023, 28% of workers in the United States and approximately 27% of workers in Japan had adopted remote work (Barrero et al. [1]; Ministry of Land [2]). Other surveys in 34 countries found that 25.6% of workers were engaged in hybrid work and 7.9% were working exclusively remotely as of April–May 2023, indicating that remote work has become an established mode of work in the post-pandemic era (Aksoy et al. [3]).

However, much of the existing research on remote work has focused on comparing productivity under remote versus on-site (or hybrid) arrangements. These macro-level studies document overall productivity differences across work arrangements but provide limited insight into within-team dynamics in remote settings. In particular, relatively few studies have directly examined whether peer effects—the influence of coworkers on an individual’s productivity—occur in fully remote teams. The limited evidence to date suggests that the productivity spillovers well documented in co-located offices may be muted or absent when employees work remotely. For example, Frakes and Wasserman [4] found that knowledge sharing among U.S. patent examiners boosted output only when peers worked in the same physical location, with this peer effect vanishing once examiners telecommuted. Similarly, Emanuel et al. [5] observed that engineers received substantially more feedback from colleagues in the same building than from those in different locations—a benefit that disappeared in a remote setting. Moreover, broad analyses indicate that remote work can sometimes reduce overall productivity by 12–20%, partly due to changes in worker interactions such as increased coordination delays and fewer spontaneous exchanges. Consistent with this view, Yang et al. [6] show that shifting to company-wide remote work reduced interpersonal connectivity and cross-team collaboration within a large technology firm. Taken together, these findings highlight an important gap: it remains unclear whether coworker-induced productivity gains can materialize when all team members are geographically dispersed, and if so, what mechanisms might enable such peer effects in fully remote settings.

By contrast, there is abundant evidence of peer effects and productivity spillovers in traditional in-person workplaces. Classic studies have shown that having high-performing or experienced colleagues can significantly raise an individual’s output. For example, Mas and Moretti [7] demonstrated that supermarket cashiers increased their checkout speed when more productive peers were present, highlighting the role of peer pressure. Likewise, Jackson and Bruegmann [8] and Bandiera et al. [9] found that employees working alongside particularly skilled or veteran coworkers achieved higher productivity, consistent with learning and knowledge spillover mechanisms. These findings underscore that coworker interactions in shared physical environments can enhance individual performance through both motivational pressure and on-the-job learning.

To frame how such peer effects might operate in remote teams, we draw on transactive memory system (TMS) theory. A TMS refers to a team’s shared understanding of “who knows what,” allowing members to locate and leverage each other’s expertise efficiently (Wegner [10]; Liang et al. [11]). In teams with a well-developed TMS, newcomers can more easily identify knowledgeable colleagues and obtain targeted guidance, which may enhance productivity even in the absence of physical proximity. While TMS and related mechanisms have been shown to matter in co-located teams, it remains an open question whether and how these mechanisms function in fully remote environments.

In light of this gap, this study examines whether working with more experienced teammates causally affects individual performance in a fully remote work setting. We use longitudinal personnel data from a Japanese company that has operated with an entirely remote workforce since its inception. New hires in this firm are effectively randomized into teams upon joining, which we exploit as a quasi-natural experiment to identify peer effects on productivity. Using this setting, we estimate the causal impact of team composition—particularly teammates’ average tenure and prior productivity—on individual output. We further examine heterogeneity by worker experience and explore whether patterns of intra-team communication are associated with productivity spillovers.

In preview, our analysis shows that having more experienced teammates significantly increases individual productivity in a fully remote setting, whereas having higher-prior-performance teammates does not yield comparable benefits. The productivity gains from experienced teammates are especially pronounced for workers with short tenure. By contrast, neither high coworker productivity nor a high volume of communication is associated with higher individual output, and for some experienced workers, lower communication intensity appears more conducive to productivity. These findings suggest that experience-based knowledge sharing, rather than peer pressure or frequent interaction per se, plays a central role in shaping productivity in remote teams.

The remainder of the paper is organized as follows. Section 2 reviews the related literature. Section 3 describes the data and empirical methodology. Section 4 presents the results, and Section 5 discusses the implications and concludes.

## Literature review

Research on productivity under remote work arrangements is growing, but a clear consensus has yet to emerge. Bloom et al. [12] conducted a randomized controlled trial on call-center employees in China and reported that working from home increased productivity by around 13%. Other studies (e.g., Angelici and Profeta [13]; Choudhury et al. [14]) similarly find that remote work and flexible working arrangements can improve worker productivity. On the other hand, Gibbs et al. [15], Emanuel and Harrington [16], and Morikawa [17] reported that productivity under remote work fell by 12–20%. Gibbs et al. [15] suggest this decline is due to increased coordination tasks and online meetings reducing uninterrupted work time, whereas Emanuel and Harrington [16] attribute it to lower-quality workers entering the remote workforce.

It has also been noted that productivity in remote work varies with the nature of the task and the worker’s experience with remote work. Dutcher [18] found that the impact of working from home depends on task type: it boosted productivity by 11–20% for creative tasks but reduced it by 6–10% for monotonous tasks. Barrero et al. [1] and Okubo et al. [19] report that productivity does not decline—and may even improve—once workers have sufficient experience with remote work. Gibbs et al. [15] and Morikawa [17] make similar points.

Another line of research concerns *transactive memory systems.* Wegner [10] introduced TMS as a collective memory structure in which group members not only store knowledge individually but also maintain awareness of “who knows what.” Liang et al. [11] extended this concept to work teams and demonstrated experimentally that groups developing a TMS outperformed those that did not. Later work (Lewis [20]) confirmed that strong TMS improves coordination and team outcomes. In our context, we interpret teams with high average tenure as more likely to have developed a robust TMS, which can lower newcomers’ search costs for information and enhance their productivity. There is extensive research on peer effects among coworkers and between supervisors and subordinates in traditional settings. Falk and Ichino [21], Bandiera et al. [22], and Mas and Moretti [7] showed that peer pressure among colleagues positively affects productivity, especially for low-skilled workers in routine tasks. Many studies have also documented peer effects among highly skilled professionals. For example, Arcidiacono et al. [23], Song et al. [24], Chan et al. [25], De Grip and Sauermann [26,27], and Bandiera et al. [9] report that employees’ productivity improves when they work with highly capable and productive coworkers, attributing this to knowledge spillovers. Moreover, De Grip et al. [27] and Jackson and Bruegmann [8] showed that working with experienced coworkers positively impacts a worker’s productivity. Similarly, some studies have found spillovers between supervisors and subordinates:Lyle and Smith [28] and Lazear et al. [29] found that subordinates paired with high-performing bosses improve their own performance and have higher promotion rates. In summary, numerous studies indicate positive peer effects in co-located work environments, both among coworkers and between supervisors and subordinates.

Apart from mechanisms like peer pressure and “learning by watching,” many studies have highlighted the importance of communication for peer effects. This suggests that knowledge transfer often occurs through structured or informal communication among coworkers. Sandvik et al. [30] conducted an RCT in a sales company to test interventions that encourage communication-driven spillovers. They compared groups with different approaches: one encouraged employees to actively share know-how and socialize (e.g., have lunch together), another provided financial incentives for information sharing, and a third combined both. They found that the group encouraged to share knowledge and socialize had higher productivity, and this effect was long-lasting. This implies that reducing the “initial cost” of communication (i.e., the hesitation or uncertainty about asking questions) is crucial for facilitating knowledge sharing. Other studies echo this point (Catalini [31]; Cai and Szeidl [32]; Hasan and Koning [33]; Boudreau et al. [34]; Battiston et al. [35]; Englmaier et al. [36]).

On the other hand, research on peer effects in remote work environments is still in early stages, and many questions remain regarding the magnitude of effects and the mechanisms involved. Emanuel et al. [5] found that engineers with coworkers in the same building received 22% more online feedback than those with coworkers in different locations, but this difference vanished under remote work—highlighting the importance of physical proximity. Frakes and Wasserman [4] likewise reported that a one standard deviation increase in a coworker’s grant rate raised the grant rate of a junior patent examiner by 0.15 standard deviations, but this peer effect disappeared when examiners worked remotely. In academia, findings are mixed: Azoulay et al. [37] and Waldinger [38] found that intellectual closeness mattered more than physical proximity for knowledge spillovers, while Catalini [31] and Boudreau et al. [34] pointed to dramatically higher collaboration rates when researchers share the same location. Several studies suggest that communication and collaboration are key for spillovers in remote work (Atkin et al. [39]; Yang et al. [6]; Garro-Abarca et al. [40]; Jarrahi and Sawyer [41]). Kirkman et al. [42], Eisenberg et al. [43], and Leenders et al. [44] concluded that in virtual teams, both excessive and insufficient communication can hurt performance, and it remains unclear whether communication quality or quantity is more important.

As noted, while many studies examine the benefits of working with talented or experienced colleagues in co-located settings, relatively little research has focused on peer effects among coworkers in fully remote environments. Frakes and Wasserman [4] and Emanuel et al. [5] show that productivity spillovers observed in person tend to disappear under remote work arrangements. However, these studies were conducted during the sudden and large-scale transition to remote work during the COVID-19 pandemic and focused on specific occupations and organizations, limiting their external validity. Moreover, because they rely on comparisons between in-person and remote settings, they do not establish whether peer effects can arise among workers who are fully remote from the outset. In addition, although communication and collaboration are often viewed as central mechanisms for productivity spillovers in remote teams, existing research provides little guidance on which aspects of communication—such as quantity versus quality—are most relevant.

Taken together, the existing literature highlights well-established peer effects and productivity spillovers in co-located work environments, while leaving important questions unanswered in the context of fully remote work. In particular, it remains unclear whether peer effects can emerge among workers who are geographically dispersed from the outset, which types of coworkers matter most for individual performance, and through which mechanisms productivity spillovers may operate in remote teams. To address these unresolved issues, the next section develops a theoretical framework based on transactive memory system theory and the peer effects literature, and derives a set of testable hypotheses regarding how teammate experience, coworker productivity, and interaction patterns shape individual performance in fully remote settings.

### Theoretical framework and hypotheses

Building on the literature above, we develop three hypotheses about how experienced teammates affect individual productivity in a fully remote work setting. Prior research on peer effects indicates that coworkers can significantly influence each other’s performance through mechanisms of social pressure, learning, and knowledge sharing. In remote teams, where face-to-face contact is absent, these mechanisms may operate differently, but we expect that experienced colleagues still provide important productivity benefits. We outline our hypotheses below, grounded in relevant theory and empirical findings.

Hypothesis 1 (H1): Having more experienced teammates increases an individual’s productivity. This hypothesis follows from evidence of positive peer effects in traditional workplaces and the knowledge-transfer advantages of experienced coworkers. Economists have documented that introducing high-performing, experienced workers into a team can raise the productivity of others through peer influence. For example, Mas and Moretti [7] found strong productivity spillovers when highly productive personnel were added to a shift of grocery workers. Similarly, Bandiera et al. [9] showed that fruit pickers worked about 10% faster when paired with a friend who was more able (productive) than themselves. These studies suggest that experienced or high-ability teammates set performance norms and directly or indirectly encourage greater effort from their peers. In addition to social pressure, experienced teammates are likely to possess valuable task knowledge that can be shared. According to transactive memory theory, team members develop a shared “who knows what” knowledge system (Wegner [10]) and draw on each other’s expertise. Empirical work has linked such collective knowledge systems to better group performance. Thus, both social influence and knowledge-sharing perspectives predict that working with more experienced colleagues will improve an individual’s output. We therefore hypothesize that a worker’s productivity rises with the average experience level of their teammates (H1).

Hypothesis 2 (H2): The productivity gains from experienced teammates are larger for less experienced (junior) workers than for more experienced workers. We posit a moderating effect of the focal worker’s own experience. Less experienced employees have greater scope for improvement and more to learn, so they should benefit disproportionately from teammates who can offer guidance, expertise, and norms of high performance. In contrast, veteran employees might already possess similar knowledge or may be less impressionable to peer influence. This idea is consistent with social learning arguments and findings in the peer-effect literature. For instance, Bandiera et al. [9] observed that in a workplace setting, employees who were less able than their friends significantly increased their productivity when a more able friend was present. In essence, junior workers may “borrow” expertise and best practices from seasoned colleagues, leading to larger relative gains in productivity. Therefore, we expect the positive effect of experienced teammates to be more pronounced for relatively inexperienced workers (H2).

Hypothesis 3 (H3): The positive impact of experienced teammates on productivity is stronger when the teammates have a history of frequent interaction (greater familiarity). Here we consider the role of team familiarity and repeated collaboration. Prior research suggests that peer effects are not uniform – they intensify when coworkers regularly interact. Mas and Moretti [7] found that workers responded more strongly to high-productivity peers with whom they frequently interacted, implying that familiarity or repeated contact amplifies peer influence. Theoretically, this can be explained by the development of transactive memory and coordination routines over time. Through repeated interaction, team members learn “who knows what” and how to work together efficiently (Wegner [10]), leading to a shared knowledge repository and better coordination. Liang et al. [11] provide empirical support for this: groups that trained together (developing shared memory of each other’s expertise) outperformed those that did not, underscoring the performance benefits of established team knowledge systems. In a remote work context, building familiarity might require deliberate communication, but the underlying premise remains that teammates who have collaborated more often can transfer know-how and tacit knowledge more effectively. Accordingly, we hypothesize that an experienced teammate’s positive effect on an individual’s productivity will be amplified when that individual has had more frequent or longer-term interactions with the teammate (H3).

## Data and methods

### Overview of Caster Inc. and analytical sample

The company has operated with a fully remote workforce since its inception; as of March 2025, it employed approximately 900 people across all 47 prefectures of Japan and in 23 other countries. This study analyzes employees in the CASTER BIZ department. We use de-identified individual-level administrative and personnel records covering April 2022 to March 2023.

New hires at Caster undergo a six-month on-the-job training (OJT) period, during which they often do not serve clients independently. To ensure that our sample consists of workers fully engaged in client-facing tasks, we exclude all employee-month observations where tenure was less than six months. The final analytical sample includes 977 employee-month observations. This dataset reflects the full population of employees in the CASTER BIZ department who met the inclusion criteria during the study period.

### Data collection procedures

Access and permissions. Data were obtained from Caster Inc. under a formal data use agreement for academic research. All data were anonymized prior to analysis: direct identifiers were removed or masked, and each employee was assigned a unique, non-identifiable ID. Access to the raw data was limited to the research team.

Data sources. The dataset integrates three internal systems: (1) the human resources management system; (2) operational performance databases; and (3) Slack communication logs. The HR system provided demographic and employment data; operational databases provided monthly client assignment counts and work-hour records; and Slack logs provided internal communication volume.

Data extraction and processing. Monthly records from each source were extracted for April 2022 through March 2023, cleaned, checked for consistency, and merged into an employee-month panel using unique employee identifiers. Observations with tenure under six months were excluded to remove the training period.

Variable construction. Productivity is measured by the number of unique clients handled per employee per month, recorded automatically in the client management system. Communication volume is measured by the number of internal Slack messages per employee per month (excluding client-facing messages). Team-level variables (e.g., average tenure, productivity, and communication volume) are computed as the average for other team members, excluding the focal individual. “Top” and “bottom” categories for experience or communication are defined using the top and bottom 25% of the relevant distributions. All definitions and cutoffs were specified prior to empirical analysis.

### Institutional setting

Caster Inc. provides online “secretary” services. Customers who contract with Caster can request various back-office tasks—such as secretarial work, human resources, accounting, and general affairs—from online secretaries (Caster employees) for up to 30 hours per month. Clients primarily submit requests via online chat tools (e.g., Slack or Microsoft Teams), and the online secretary carries out the requested work.

Employees hired as online secretaries undergo a six-month on-the-job training period, after which they are assigned to clients. The company follows a “same work, same pay” principle: an online secretary’s monthly salary is determined by the number of clients they serve, regardless of their previous salary. There is no minimum quota of clients; each employee can decide how many clients to take on.

New online secretaries are allocated by a mechanical round-robin rule: the first newcomer goes to Team A, the next to Team B, then Team C, and so on, cycling back to Team A. Neither managers nor workers can deviate from this rotation—there is no screening, swapping, or requesting of teams—so neither managers nor new hires have discretion over the destination team. Because the order of arrivals is orthogonal to team characteristics, the resulting placement is as-if randomized across teams within each join month. We exploit this institutional feature as a quasi-natural experiment in our analysis.

Each team is designed to consist of 4–12 online secretaries and exactly one designated team leader. Team leaders do not have their own client quotas and there are no additional performance incentives based on team outcomes. When constructing our analytical sample, we verified that all teams in the study period followed this “single-leader” structure. Any teams that would not have met this criterion (e.g., teams without a leader or with multiple leaders) would have been excluded, although in practice no such cases occurred in our dataset.

### Outcome and key variables

The primary outcome variable is individual productivity, proxied by the number of clients handled in a given month. This is a reasonable proxy because the firm operates under standardized working hours (9:00–17:00 on weekdays), and each client contract entitles the client to 30 hours of monthly support. The more clients a worker handles, the greater the implied output, assuming roughly comparable working hours across employees.

Key explanatory variables include the lagged average productivity and average tenure of teammates (excluding the individual), and indicators for whether a worker’s team falls into the top or bottom quartile of experience or communication intensity. Communication volume is derived from internal Slack messages among employees.

### Empirical strategy

To estimate the impact of team characteristics on individual productivity, we estimate fixed-effects panel models that control for time-invariant individual heterogeneity and common time shocks. The key independent variable is the one-month lag of teammates’ average productivity (or experience), excluding the focal individual. Control variables include age, gender, tenure, team size, working time, and lagged average team Slack activity (excluding the individual). All models include individual, team, and calendar-month fixed effects. Standard errors are clustered at the individual and team levels.

where subscripts i, j, and t denote individual, team, and month, respectively. $Y_{ijt}$ is the outcome variable, defined as the natural logarithm of the number of clients that individual 𝑖 in j team handles at time t. ${\overset{―}{X}}_{-ijt-\text{1}}$ is the average number of clients handled by team j at time t−1, excluding individual 𝑖. $\eta^{\prime }{𝐙}_{ijt}$ is a vector of control variables, including individual i’s and team j’s characteristics (age, female, team size, tenure, working time and post team mean at time t−1, excluding i). $\gamma_{i}$ is an individual fixed effect capturing time-invariant characteristics of worker 𝑖, $\delta_{j}$ is a team fixed effect, $\zeta_{t}\;$is a month fixed effect, and $\varepsilon_{ijt}$ is an error term.

When estimating peer effects, two issues must be addressed. First, we must account for potential endogeneity related to individual and team characteristics. For example, unobserved abilities of individuals or their coworkers could influence productivity, biasing the estimates. We mitigate this by including individual and team fixed effects in the model. Second, there is the reflection problem (Manski [45]), which complicates causal interpretation because coworkers influence each other simultaneously.

To address the reflection problem, Yeung and Nguyen-Hoang [46]suggest two approaches: using instrumental variables in a two-stage least squares framework, or using lagged peer variables. Following prior studies (Yeung & Nguyen-Hoang [46]; An et al. [47]; Cassar and Ko [48]), we use the one-month lag of peer average productivity (team average clients excluding 𝑖) to mitigate simultaneity.

### Robustness and randomization checks

We leverage the firm’s mechanical team assignment system (round-robin by hire order) to claim as-if random assignment of new hires to teams. To support this claim, we conduct ANOVA tests and permutation-based randomization inference. We further test robustness by including team-by-entry-month fixed effects and alternative peer exposure definitions (e.g., indicator for at least one top-quartile peer). Results are consistent across specifications, confirming the validity of our identification strategy.

### Descriptive statistics

Table 1 presents basic summary statistics for the sample. Detailed descriptions of variables are provided in Table 2.

**Table 1:** Basic statistics.

	N	Mean	Min	Max	SD
Number of clients	977	8.191	0.4	27.7	3.847
Age	977	36.377	26	52	6.911
Tenure	977	35.853	7	83	19.312
Female	977	0.976	0	1	0.152
Team size	977	6.623	3	12	3.297
Working Time	977	160.245	158.5	162.4	2.021
Customer Team Mean	898	8.189	2.2	13.4	1.523
Post Team Mean	898	434.367	107.5	1,403.00	148.406
Top Tenure	977	0.237	0	1	0.426
Tenure bottom	977	0.271	0	1	0.445
Tenure Under	977	0.238	0	1	0.426
Tenure Middle	977	0.253	0	1	0.435
Top Pro	977	0.232	0	1	0.423
Pro bottom	977	0.276	0	1	0.447
Pro Under	977	0.253	0	1	0.435
Pro Middle	977	0.238	0	1	0.426
Top25 Comm Volume	898	0.293	0	1	0.455
bottom25 Comm Volume	898	0.356	0	1	0.479
Top Tenure in Team	977	0.850	0	1	0.358
Top Pro in Team	977	0.704	0	1	0.457

**Table 2:** Variable description.

Variables	Description
Number of clients	Number of clients handled per worker per month
Age	Age
Tenure	Number of months enrolled
Female	Dummy variable (women = 1)
Team size	Number of team members excluding individual i
Working Time	Hours worked per month, in minutes
Customer Team Mean	Average number of customers handled by team j in period t-1, excluding individual i
Post Team Mean	Average number of posts by team j in period t-1, excluding individual i
Top Tenure	Dummy variable equal to 1 if the average months of tenure of the team members (excluding individual i) is in the top 25% of the distribution.
Tenure bottom	Dummy variable equal to 1 if the average months of tenure of the team members (excluding individual i) is in the bottom 25% of the distribution.
Tenure Under	Dummy variable equal to 1 if the average months of tenure of the team members (excluding individual i) is in the bottom 25–50% of the distribution (i.e., the second-lowest quartile).
Tenure Middle	Dummy variable equal to 1 if the average months of tenure of the team members (excluding individual i) is in the top 25–50% of the distribution (i.e., the second-highest quartile).
Top Pro	Dummy variable equal to 1 if the average of productivity(the number of client) of the team members (excluding individual i) is in the top 25% of the distribution.
Pro bottom	Dummy variable equal to 1 if the average of productivity(the number of client) of the team members (excluding individual i) is in the bottom 25% of the distribution.
Pro Under	Dummy variable equal to 1 if the average of productivity(the number of client) of the team members (excluding individual i) is in the bottom 25–50% of the distribution (i.e., the second-lowest quartile).
Pro Middle	Dummy variable equal to 1 if the average of productivity(the number of client) of the team members (excluding individual i) is in the top 25–50% of the distribution (i.e., the second-highest quartile).
Top25 Comm Volume	Dummy variable equal to 1 if the average volume of texts(via Slack) of the team members (excluding individual i) is in the top 25% of the distribution.
bottom25 Comm Volume	Dummy variable equal to 1 if the average volume of texts(via Slack) of the team members (excluding individual i) is in the bottom 25% of the distribution.
Top Tenure in Team	Dummy variable equal to 1 if at least one other member of i’s team is Top Tenure (=1)
Top Pro in Team	Dummy variable equal to 1 if at least one other member of i’s team is Top Pro (=1)

### Ethics statement

This study was an observational analysis of retrospective, fully anonymized business records from company employees. In accordance with institutional guidelines, formal ethics committee (Institutional Review Board) review was not required since the research did not involve any identifiable personal data or direct interaction with human participants. The dataset did not include any minors, and informed consent was not required because all records were pre-existing and de-identified prior to analysis. The study was conducted in compliance with all relevant ethical standards for research using anonymized data.

## Results

### Main estimation

Using the model above, we estimate the effect of team productivity on individual productivity. The results are shown in Table 3.

**Table 3:** Main results(dependent variable: log number of clients).

	(1)	(2)	(3)
	−0.016 (0.013)	−0.059 (0.019)**	−0.040 (0.021)
Customer Team Mean			
Customer Team Mean × Top Tenure		0.122 (0.026)***	
Customer Team Mean × Top Pro			0.063 (0.044)
Individual FE	YES	YES	YES
Team FE	YES	YES	YES
Month FE	YES	YES	YES
Controls	YES	YES	YES
obs.	786	784	785
R2 Adj	0.521	0.771	0.770
Cluster	id + team	id + team	id + team

*Notes*: The variable Customer Team Mean × Top Tenure indicate that the average tenure of the team is in the top 25% of all teams. The variable Customer Team Mean × Top Pro indicate that the average number of customers handled by the team is in the top 25% of all teams. Significance Codes: 0 ‘***’ 0.001 **’ 0.01 ‘*’ 0.05 ‘.’ 0.1

We find no evidence that being on a team with high average productivity raises an individual’s own productivity. Even for teams with top-quartile average productivity, we find no significant effect on individual output. However, when team members are highly experienced (teams with top-quartile average tenure), the productivity of workers on those teams increases by about 12.2%. In a fully remote work setting, we do not observe a peer effect from working with highly productive (high-output) coworkers, but we do find evidence of a peer effect from working with experienced coworkers.

### Subsample estimation

To examine how peer effects vary with tenure, we split the sample by employee tenure quartiles and re-estimate the model for each subgroup (see Table 4).

**Table 4:** Sub sample results.

	Tenure bottom	Tenure Under	Tenure Middle	Tenure Top
	(4)	(5)	(6)	(7)
Customer Team Mean	−0.081 (0.039) *	−0.045 (0.031)	−0.006 (0.033)	−0.015 (0.049)
Customer Team Mean × Top Tenure	0.262 (0.074) ***	0.086 (0.040) *	0.026 (0.034)	0.009 (0.029)
Individual FE	YES	YES	YES	YES
Team FE	YES	YES	YES	YES
Month FE	YES	YES	YES	YES
controls	YES	YES	YES	YES
obs.	184	162	175	176
R2 Adj	0.475	0.777	0.341	0.621
Cluster	id + team	id + team	id + team	id + team

*Notes*: The outcome variable is the logarithm of the number of clients. Workers in the first quartile were classified as “Tenure bottom”, workers in the second quartile as “Tenure under”, workers in the third quartile as “Tenure middle”, and workers in the top 25 percentile as “Tenure top”. Significance Codes: 0 ‘***’ 0.001 **’ 0.01 ‘*’ 0.05 ‘.’ 0.1.

The subsample results indicate that productivity increases by approximately 26.2% for workers in the shortest tenure group (first quartile), and by about 8.6% for those in the second quartile (tenure between the 25th and 50th percentile). These findings are consistent with Frakes and Wasserman [4], who observed peer effects for employees with less than two years at the company, with the effect diminishing as tenure increased.

By contrast, when we split the sample by individual productivity levels, we do not find statistically significant peer effects in any productivity quartile (see Table 5).

**Table 5:** The effect of joining to top pro team(divide into quartile of the tenure).

	Tenure bottom	Tenure Under	Tenure Middle	Tenure Top
	(8)	(9)	(10)	(11)
Customer Team Mean	−0.056 (0.051)	0.039 (0.027)	−0.024 (0.037)	−0.083 (0.062)
Customer Team Mean × Top Pro	0.104 (0.115)	−0.071 (0.074)	0.018 (0.031)	0.062 (0.055)
Individual FE	YES	YES	YES	YES
Team FE	YES	YES	YES	YES
Month FE	YES	YES	YES	YES
controls	YES	YES	YES	YES
obs.	183	161	174	175
R2 Adj	0.478	0.777	0.338	0.692
Cluster	id + team	id + team	id + team	id + team

*Notes*: The outcome variable is the logarithm of the number of clients. The variable Customer Team Mean × Top Pro indicate that the average number of customers handled by the team is in the top 25% of all teams. Workers in the first quartile were classified as “Tenure bottom”, workers in the second quartile as “Tenure under”, workers in the third quartile as “Tenure middle”, and workers in the top 25 percentile as “Tenure top”. Significance Codes: 0 ‘***’ 0.001 **’ 0.01 ‘*’ 0.05 ‘.’ 0.1.

Additionally, we examined whether having exceptionally productive coworkers (top 25% individually) influences outcomes. We conducted subsample analyses by worker productivity and by tenure for those workers whose teams included highly productive peers, but found no significant effects in those cases either (see Tables 6 and 7).

**Table 6:** The effect of joining to top tenure team (divide into quartile of the productivity).

	Pro bottom	Pro Under	Pro Middle	Pro Top
	(12)	(13)	(14)	(15)
Customer Team Mean	−0.101 (0.044)	−0.094 (0.053)	0.066 (0.035)	0.002 (0.014)
Customer Team Mean × Top Tenure	0.109 (0.167)	0.129 (0.135)	0.017 (0.021)	0.008 (0.028)
Individual FE	YES	YES	YES	YES
Team FE	YES	YES	YES	YES
Month FE	YES	YES	YES	YES
controls	YES	YES	YES	YES
obs.	180	198	191	183
R2 Adj	0.776	0.386	0.276	0.645
Cluster	id + team	id + team	id + team	id + team

*Notes*: The outcome variable is the logarithm of the number of clientss. The variable Customer Team Mean × Top Tenure indicate that the average tenure of team members is in the top 25% of all teams. The productivity of workers in the first quartile were classified as “Pro bottom”, workers in the second quartile as “Pro under”, workers in the third quartile as “Pro middle”, and workers in the top 25 percentile as “Pro Top”. Significance Codes: 0 ‘***’ 0.001 **’ 0.01 ‘*’ 0.05 ‘.’ 0.1

**Table 7:** The effect of joining to top pro team(divide into quartile of the productivity).

	Pro bottom	Pro Under	Pro Middle	Pro Top
	(16)	(17)	(18)	(19)
Customer Team Mean	−0.211 (0.094)	−0.025 (0.035)	0.036 (0.026)	0.004 (0.017)
Customer Team Mean × Top Pro	0.109 (0.094)	0.041 (0.058)	−0.084 (0.053)	0.018 (0.031)
				
Individual FE	YES	YES	YES	YES
Team FE	YES	YES	YES	YES
Month FE	YES	YES	YES	YES
control	YES	YES	YES	YES
obs.	180	198	191	183
R2 Adj	0.776	0.386	0.276	0.645
Cluster	id + team	id + team	id + team	id + team

*Notes*: The outcome variable is the logarithm of the number of clients. The variable Customer Team Mean × Top Pro indicate that the average number of customers handled by the team is in the top 25% of all teams. The productivity of workers in the first quartile were classified as “Pro bottom”, workers in the second quartile as “Pro under”, workers in the third quartile as “Pro middle”, and workers in the top 25 percentile as “Pro Top”. Significance Codes: 0 ‘***’ 0.001 **’ 0.01 ‘*’ 0.05 ‘.’ 0.1

These results suggest that in a remote work environment, working with experienced coworkers (as opposed to simply high-output coworkers) is what boosts individual productivity—particularly for employees who are new to the company.

### The effect of communication

**Fig 1:**
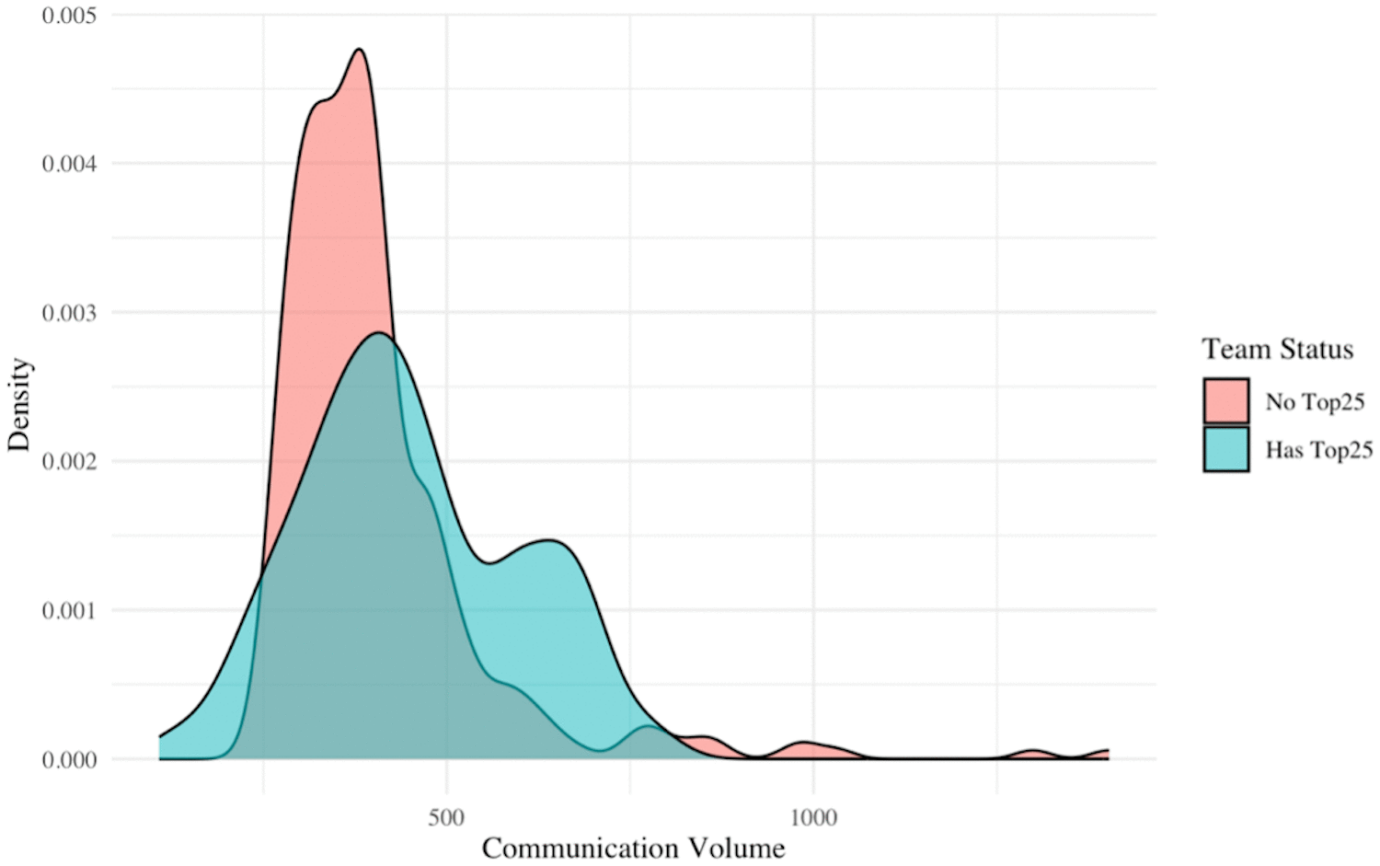
Comparison of communication volume between high-tenure teams and other teams. This figure shows the density function of communication volume on Slack between employees belonging to Top Tenure and those who do not. The former are shown in blue, the latter in red.

We next explore whether the volume of communication within a team influences individual productivity. The employees covered in this study do not communicate via email, hold only one company-wide meeting per month, and conduct nearly all communication through Slack. This study measures only the volume of internal Slack messages, excluding client-facing messages. We define this as communication volume and aim to clarify the effect of communication volume within teams on individual employee productivity. We compare teams with high average tenure (where we found peer effects) to other teams in terms of communication volume. Fig 1 shows that teams with top-quartile average tenure actually have a lower volume of communication than other teams. A Wilcoxon rank-sum test confirms a statistically significant difference (p = 0.001) in communication volume: high-tenure teams communicate less than other teams. This suggests that a high amount of intra-team communication might not be necessary for peer productivity effects to occur.

**Fig 2:**
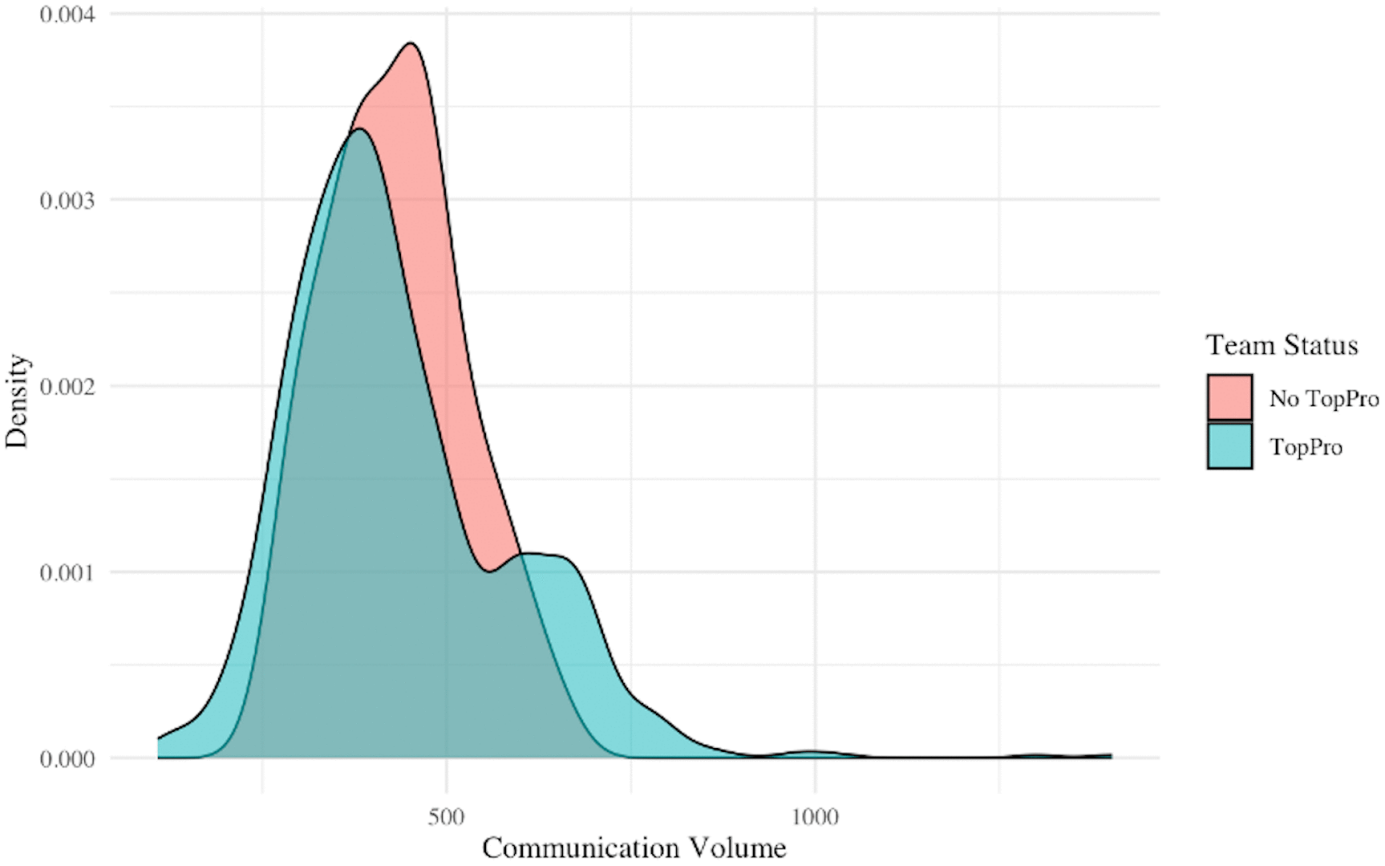
Comparison of communication volume between high-productivity teams and other teams. This figure shows the density function of communication volume on Slack between employees belonging to Top Pro and those who do not. The former are shown in blue, the latter in red.

We also compared communication levels between teams with high versus low average productivity, and found no significant difference (see Fig 2). A Wilcoxon rank-sum test indicates no statistical difference in the volume of communication between the two (P-value = 0.486).

Interestingly, when we segment by worker tenure and productivity, we find that long-tenured workers and already high-productivity workers tend to perform better when they are on teams with low communication levels. Table 8 and 9 present these results. In other words, experienced and highly productive employees seem to benefit, at least to some extent, from being in a quieter team environment.

**Table 8:** The effect of communication on productivity(divide into quartile of the tenure).

	Tenure bottom	Tenure Under	Tenure Middle	Tenure Top
	(20)	(21)	(22)	(23)
Customer Team Mean	−0.059 (0.044)	0.024 (0.022)	0.018 (0.016)	−0.076 (0.040)
Customer Team Mean × Top25 Comm Volume	−0.006 (0.044)	0.044 (0.063)	−0.003 (0.026)	−0.011 (0.028)
Individual FE	YES	YES	YES	YES
Team FE	YES	YES	YES	YES
Month FE	YES	YES	YES	YES
controls	YES	YES	YES	YES
obs.	184	162	175	176
R2 Adj	0.495	0.781	0.362	0.632
Cluster	id + team	id + team	id + team	id + team
	**Tenure** **bottom**	**Tenure** **Under**	**Tenure** **Middle**	**Tenure** **Top**
	**(24)**	**(25)**	**(26)**	**(27)**
Customer Team Mean	−0.027 (0.022)	0.005 (0.011)	−0.009 (0.012)	−0.098 (0.032)*
Customer Team Mean × bottom25 Comm Volume	−0.023 (0.034)	0.006 (0.027)	0.033 (0.026)	0.090 (0.028)*
Individual FE	YES	YES	YES	YES
Team FE	YES	YES	YES	YES
Month FE	YES	YES	YES	YES
control	YES	YES	YES	YES
obs.	184	162	175	176
R2 Adj	0.442	0.767	0.351	0.616
Cluster	id + team	id + team	id + team	id + team

*Notes*: The outcome variable is the logarithm of the number of clientss. The variable Customer Team Mean × Top25_Comm_Volume indicate that the communication volume of team is at the top 25%, and The variables Customer Team Mean × bottom25_Comm_Volume indicate that the communication volume of team is at the bottom 25%. Significance Codes: 0 ‘***’ 0.001 **’ 0.01 ‘*’ 0.05 ‘.’ 0.1.

**Table 9:** The effect of communication on productivity(divide into quartile of the productivity).

	Pro bottom	Pro Under	Pro Middle	Pro Top
	(28)	(29)	(30)	(31)
Customer Team Mean	−0.106 (0.035)*	−0.007 (0.020)	0.015 (0.013)	−0.001 (0.009)
Customer Team Mean × Top25 Comm Volume	−0.046 (0.051)	−0.004 (0.035)	0.009 (0.021)	0.019 (0.024)
Individual FE	YES	YES	YES	YES
Team FE	YES	YES	YES	YES
Month FE	YES	YES	YES	YES
control	YES	YES	YES	YES
obs.	180	198	191	183
R2 Adj	0.766	0.325	0.203	0.395
Cluster	id + team	id + team	id + team	id + team
	**Pro** **bottom**	**Pro** **Under**	**Pro** **Middle**	**Pro** **Top**
	**(32)**	**(33)**	**(34)**	**(35)**
Customer Team Mean	−0.104 (0.042)*	−0.045 (0.032)	0.034 (0.011)*	0.032 (0.009)*
Customer Team Mean × bottom25 Comm Volume	0.059 (0.034)	0.019 (0.025)	−0.024 (0.019)	0.062 (0.028)*
Individual FE	YES	YES	YES	YES
Team FE	YES	YES	YES	YES
Month FE	YES	YES	YES	YES
control	YES	YES	YES	YES
obs.	180	198	191	183
R2 Adj	0.759	0.342	0.226	0.405
Cluster	id + team	id + team	id + team	id + team

*Notes*: The outcome variable is the logarithm of the number of clientss. The variable Customer Team Mean × Top25_Comm_Volume indicate that the communication volume of team is at the top 25%, and The variables Customer Team Mean × bottom25_Comm_Volume indicate that the communication volume of team is at the bottom 25%. Significance Codes: 0 ‘***’ 0.001 **’ 0.01 ‘*’ 0.05 ‘.’ 0.1.

### Random assignment check

We exploit the random assignment of new employees to teams to help identify peer effects. A potential concern is that team assignments might be endogenous. To verify the randomness of team assignment, we conducted an ANOVA comparing characteristics of employees across teams.

The ANOVA results (see Table 10) indicate that only one characteristic—age—differs significantly across teams (at the 5% level).

**Table 10:** The ANOVA test results.

	F-value	P-Value
Tenure	2.078	0.150
Female	2.324	0.128
Working Time	7.271	0.437
Education	0.007	0.935

*Notes*: Significance Codes: 0 ‘***’ 0.001 **’ 0.01 ‘*’ 0.05 ‘.’ 0.1

We include age as a control in the regressions, which should account for this slight discrepancy. To address cohort-specific baseline sorting, we add team × entry-month fixed effects; results are virtually unchanged relative to the baseline, supporting the view that initial assignment is not driving our estimates. Identification then comes from within team × cohort variation over time. As shown in Table 11, the results are virtually unchanged relative to the baseline, supporting the view that initial assignment is not driving our estimates and is consistent with quasi-random assignment at entry.

**Table 11:** Robustness to team × join-month fixed effects.

	y = log(Customer Number)		
	(36)	(37)	(38)
Customer Team Mean	−0.009 (0.010)	−0.044 (0.009) **	−0.036 (0.021)
Customer Team Mean × Top Tenure		0.098 (0.016) **	
Customer Team Mean × Top Pro			0.046 (0.040)
Individual FE	YES	YES	YES
Month FE	YES	YES	YES
Join Month FE × Team FE	YES	YES	YES
controls	YES	YES	YES
obs.	786	784	785
R2 Adj	0.511	0.797	0.769
Cluster	id + team	id + team	id + team

*Notes*: All models include individual and calendar-month fixed effects. The specification in Column adds team × join-month fixed effects. Results are virtually unchanged relative to the baseline. Significance Codes: 0 ‘***’ 0.001 **’ 0.01 ‘*’ 0.05 ‘.’ 0.1

Additionally, using randomization inference, we randomly reassigned team labels within entry month 300 times while preserving team sizes and re-estimated the same specification each time. The permutation p-value for the coefficient on the lagged peer average (team mean excluding the focal individual) was 0.193 (two-sided). The observed estimate is not unusually large relative to the randomization distribution, again consistent with quasi-random assignment at entry.

### Robustness check

We conducted several additional analyses to check the robustness of our findings. First, we confirmed that there are no significant differences in observable worker attributes between teams with high vs. low average tenure, or between teams with high vs. low average productivity (see Table 12). This suggests that selection on observables is unlikely to drive our results.

**Table 12:** Attribute comparison of employees.

	Top Tenure	No TopTenure	P-value
Age	37.422	34.585	0.081
Female	0.986	0.950	0.514
Working Time	160.025	160.450	0.843
Education	14.286	14.666	0.815
Number of clients	8.328	7.366	0.269
	**Top Pro**	**No TopPro**	**P-value**
Age	36.947	36.178	0.105
Female	0.986	0.973	0.071
Working Time	160.015	160.055	0.422
Education	15.509	15.352	0.254
Tenure	35.931	35.785	0.834

We also tested alternative definitions of peer exposure. Instead of using team averages, we created indicators for whether a team contains at least one exceptionally high-performing coworker (top 25% in individual productivity) or at least one very experienced coworker (top 25% in tenure). We then examined the effects of these indicators on individual productivity (see Table 13). The results are in line with our main findings, confirming that our conclusions are robust. However, the estimated effect sizes using these indicators are somewhat larger (about a 30.1% increase for the shortest-tenure group), suggesting a possible upward bias when not fully accounting for individual and team fixed effects.

**Table 13:** Robustness check results.

	All sample			
	(39)	(40)		
Customer Team Mean	−0.103 (0.021)**	−0.198 (0.031)***		
Customer Team Mean × Top Pro in Team	0.115 (0.034)**			
Customer Team Mean × Top Tenure in Team		0.032 (0.025)		
Individual FE	NO	NO		
Team FE	NO	NO		
Month FE	YES	YES		
control	YES	YES		
obs.	864	856		
R2 Adj	0.106	0.078		
Cluster	id + team	id + team		
	**Tenure** **bottom**	**Tenure** **Under**	**Tenure** **Middle**	**Tenure** **Top**
	**(41)**	**(42)**	**(43)**	**(44)**
Customer Team Mean	−0.144 (0.057)**	−0.298 (0.073)***	−0.277 (0.095)***	−0.246 (0.104)**
Customer Team Mean × Top Pro in Team	0.301 (0.105)**	0.085 (0.067)	0.067 (0.060)	0.054 (0.045)
Individual FE	NO	NO	NO	NO
Team FE	NO	NO	NO	NO
Month FE	YES	YES	YES	YES
controls	YES	YES	YES	YES
obs.	220	197	238	175
R2 Adj	0.087	0.265	0.125	0.098
Cluster	id + team	id + team	id + team	id + team
	**Pro** **bottom**	**Pro** **Under**	**Pro** **Middle**	**Pro** **Top**
	**(45)**	**(46)**	**(47)**	**(48)**
Customer Team Mean	−0.059 (0.027)*	−0.097 (0.030)*	−0.225 (0.053)***	−0.077 (0.076)
Customer Team Mean × Top Tenure in Team	0.044 (0.060)	0.084 (0.047)	0.088 (0.062)	−0.119 (0.102)
Individual FE	NO	NO	NO	NO
Team FE	NO	NO	NO	NO
Month FE	YES	YES	YES	YES
controls	YES	YES	YES	YES
obs.	230	198	229	176
R2 Adj	0.097	0.254	0.167	0.122
Cluster	id + team	id + team	id + team	id + team

*Notes*: The outcome variable is the logarithm of the number of clientss. The variable Customer Team Mean × Top Pro in team indicate that coworkers who are at the top 25% of individual productivity exist in the same team, and The variables Customer Team Mean × Top Tenure in team indicate that coworkers who are at the top 25% of individual tenure exist in the same team. Significance Codes: 0 ‘***’ 0.001 **’ 0.01 ‘*’ 0.05 ‘.’ 0.1.

## Discussion and conclusion

### Discussion and conclusion

Our findings provide clear evidence that experienced teammates can significantly enhance individual productivity in a fully remote work setting, whereas simply working alongside highly productive coworkers does not. In particular, employees assigned to teams with high average tenure experienced substantial productivity gains, while no comparable spillover was observed from teammates’ average productivity or from higher communication volume. These results highlight the importance of experience-based mechanisms, rather than peer pressure or frequent interaction per se, in shaping productivity in remote teams.

Hypothesis 1 (H1), which predicted that working with more experienced teammates would increase individual productivity, is strongly supported by the data. Employees in teams with longer average tenure exhibited significantly higher output, especially among those with short tenure themselves. This pattern is consistent with transactive memory system (TMS) theory, which emphasizes the role of shared knowledge about “who knows what” within a team (Wegner [10]; Liang et al. [11]). In a fully remote environment, where informal observation and spontaneous interactions are limited, experienced teammates may help newcomers navigate tasks more efficiently by lowering search costs for information and providing targeted guidance. Our findings suggest that such TMS-based mechanisms can operate even in the absence of physical proximity.

By contrast, Hypothesis 2 (H2), which posited productivity spillovers from working alongside highly productive peers through peer pressure, is not supported. Unlike in co-located settings studied by Mas and Moretti [7] or Bandiera et al. [9], we find no evidence that teammates’ average productivity raises individual output in remote teams. One plausible explanation is that remote workers cannot easily monitor each other’s effort or performance, weakening social pressure. In addition, under the compensation system studied here, individual pay depends solely on personal output rather than team performance. As emphasized by Hamilton et al. [49], peer effects driven by peer pressure are more likely to arise when team-based incentives are present. In our setting, workers have little incentive to adjust effort in response to high-performing peers, which may explain the absence of peer-pressure effects.

Hypothesis 3 (H3), which emphasized knowledge spillovers mediated by interaction and communication, is also not supported in a straightforward manner. We find no evidence that higher volumes of communication enhance individual productivity, nor that working with highly productive teammates generates spillovers through frequent interaction. Instead, communication quantity appears to be a poor proxy for effective knowledge transfer in remote teams. This finding is consistent with prior work suggesting that excessive communication can crowd out focused work time (Bloom et al. [12]; Gibbs et al. [15]). At the same time, our results do not imply that communication is unimportant; rather, they suggest that the quality and targeting of interactions—potentially facilitated by experienced teammates—may matter more than sheer volume.

Importantly, our results distinguish clearly between the role of experience and the role of communication volume. While prior literature often treats communication as the primary channel of spillovers, our findings suggest that productivity gains stem from accumulated team experience rather than the sheer quantity of interaction. In other words, it is not frequent communication itself, but the presence of experienced teammates who can provide targeted and efficient guidance, that drives the observed productivity effects.

Taken together, these findings indicate that peer effects in fully remote teams differ fundamentally from those observed in co-located environments. While peer pressure and “learning by watching” play an important role in traditional workplaces, experience-based knowledge structuring appears to be the dominant mechanism in remote settings. Experienced teammates may provide just enough guidance to reduce the initial cost of asking questions for newcomers, without requiring frequent interaction. This interpretation aligns with evidence that experience is critical for sustaining productivity in remote work arrangements (Barrero et al. [1]; Morikawa [17]; Okubo et al. [19]).

Several limitations warrant discussion. First, although our fixed-effects strategy mitigates concerns about time-invariant heterogeneity, we cannot fully eliminate the possibility that unobserved time-varying characteristics influence both peer composition and productivity. Second, our analysis is based on a single fully remote organization, which may limit external validity. Third, we do not observe the content or quality of communication, preventing direct identification of knowledge-transfer channels.

In conclusion, our results show that who one works with continues to matter in remote work—but in different ways than in traditional offices. Assigning less experienced workers to teams with seasoned colleagues can yield substantial productivity gains, even in fully remote organizations. By contrast, simply grouping workers with high performers or increasing communication intensity does not generate comparable benefits. These findings suggest that effective team design in remote work should prioritize experience-based knowledge structures over peer pressure or frequent interaction.

### Practical implications

These findings provide valuable insights for managers and organizations. Working in a team with experienced coworkers can improve the productivity of remote workers, especially those who are new to the company. In contrast, for employees who are themselves experienced or highly productive, an environment with fewer distractions and less frequent communication may be more conducive to productivity.

First, managers might consider team reassignments or mentoring setups based on employee experience and performance. For example, pairing junior, less-experienced employees with veteran coworkers could help accelerate learning and boost productivity by lowering the “initial cost” of seeking help. Meanwhile, giving more autonomy and quiet space to senior or high-performing employees—rather than placing them in communication-heavy teams—could help maintain or improve their output.

More broadly, as remote work continues to evolve, it is important for organizations to identify and implement evidence-based practices to support productivity. This will require leveraging data on employee performance and collaboration from multiple companies to pinpoint what works and why. Currently, many HR decisions are guided by intuition and experience; going forward, firms should invest in collecting and analyzing employee data, developing HR professionals who can use data for strategic decisions, and building data infrastructure to facilitate evidence-based management in a remote work era.
